# Staphylococcus epidermidis strains isolated from breast milk of women suffering infectious mastitis: potential virulence traits and resistance to antibiotics

**DOI:** 10.1186/1471-2180-9-82

**Published:** 2009-05-07

**Authors:** Susana Delgado, Rebeca Arroyo, Esther Jiménez, Maria L Marín, Rosa del Campo, Leonides Fernández, Juan M Rodríguez

**Affiliations:** 1 Dpt. Nutrición, Bromatología y Tecnología de los Alimentos. Universidad Complutense de Madrid, 28040 Madrid, Spain; 2 Servicio de Microbiología, Hospital Ramón y Cajal, 28034 Madrid, Spain

## Abstract

**Background:**

Although *Staphylococcus aureus *is considered the main etiological agent of infectious mastitis, recent studies have suggested that coagulase-negative staphylococci (CNS) may also play an important role in such infections. The aims of this work were to isolate staphylococci from milk of women with lactational mastitis, to select and characterize the CNS isolates, and to compare such properties with those displayed by CNS strains isolated from milk of healthy women.

**Results:**

The milk of 30 women was collected and bacterial growth was noted in 27 of them, of which *Staphylococcus epidermidis *was isolated from 26 patients and *S. aureus *from 8. Among the 270 staphylococcal isolates recovered from milk of women with mastitis, 200 were identified as *Staphylococcus epidermidis *by phenotypic assays, species-specific PCR and PCR sequencing. They were typified by pulsed field gel electrophoresis (PFGE) genotyping. The PFGE profiles of the *S. epidermidis *strains were compared with those of 105 isolates from milk of healthy women. A representative of the 76 different PFGE profiles was selected to study the incidence of virulence factors and antibiotic resistance. The number of strains that contained the biofilm-related *ica*D gene and that showed resistance to oxacillin, erythromycin, clindamycin and mupirocin was significantly higher among the strains isolated from mastitic milk.

**Conclusion:**

*S. epidermidis *may be a frequent but largely underrated cause of infectious mastitis in lactating women. The resistance to diverse antibiotics and a higher ability to form biofilms found among the strains isolated from milk of women suffering mastitis may explain the chronic and/or recurrent nature of this infectious condition.

## Background

Mastitis is a common condition during lactation and its incidence oscillates between 5 and 33% of the lactating mothers [1,2]. The number of non-infectious mastitis that become an infectious disease is usually so high that some authors define the term "mastitis" as an infectious process of the mammary gland characterized by a variety of local and systemic symptoms [3]. However, the number of studies dealing with the microbiological aspects of human mastitis is low and the role of specific agents has yet to be described. In fact, published articles on the bacteria causing this condition are scarce and most are, at least, 10 years old [2].

Traditionally, *Staphylococcus aureus *has been considered the most common etiological agent although, unfortunately, the cases in which microbiological analyses are performed are exceptional. However, treatments with antibiotic or antifungal drugs are usually prescribed without knowing the etiology or the antibiotic susceptibility of the microorganism involved. This practice may lead to a worsening of the symptoms since strains that cause mastitis may exhibit multirresistance to drugs and/or form biofilms. Therefore, a better knowledge of the main features of the bacterial species involved in the mastitic process would represent a great advance for the design of new strategies for the prevention and/or treatment of this condition.

In a previous work, we investigated the microbial diversity of breast milk in 20 women with lactational mastitis by culture-dependent and -independent techniques [4], and observed that staphylococci, mainly *Staphylococcus epidermidis*, seem to be the major microorganisms present in breast milk of women with infectious mastitis. In recent years *S. epidermidis *has become increasingly recognized as opportunistic pathogen [5,6]. Parallel, several genetic determinants involved in mechanisms of adhesion and biofilm formation have been described in this species [7,8] while its rate of resistance to several antibiotics has increased during the last years [9-11].

In this context, the objective of the present study was to evaluate the presence of *S. epidermidis *in breast milk of women with infectious mastitis, to characterize the isolates and to compare their properties with those of strains isolated from milk of healthy women.

## Results

### Bacterial counts and identification of staphylococci in milk

Presence of staphylococci was observed in 27 of the 30 samples provided by women with lactational mastitis. In these samples, counts in Baird Parker (BP) agar plates ranged between 4.0 and 6.0 log_10 _cfu mL^-1 ^(Table 1). A total of 270 isolates were obtained from the BP plates (10 from each woman) and all of them were lysozyme-resistant, lysostaphin-sensitive, catalase-positive, Gram-positive cocci. Among these presumptive staphylococcal isolates, 200 were identified as *S. epidermidis *on the basis of biochemical tests and species-specific PCR assays. This species was present in 26 milk samples. Only 35 staphylococcal isolates belonged to the species *S. aureus *and they were obtained from milk of eight women. PCR sequencing of a 16S rDNA fragment confirmed the results. The remaining 35 isolates that gave no amplification with the multiplex PCR were further identified by 16S rDNA PCR sequencing as *Staphylococcus pasteuri *(n = 16), *Staphylococcus warneri *(n = 11) and *Staphylococcus hominis *(n = 8) (Table 1). The partial 16S rDNA sequences obtained from single isolates belonging to the species *Staphylococcus aureus *and *Staphylococcus epidermidis *were deposited in the EMBL nucleotide sequence database under accession numbers [EMBL: AM697666] and [EMBL: AM697667], respectively. Then, our attention was focused on the *S. epidermidis *isolates.

**Table 1:** Samples and isolates used in this study

Milk sample	Staphylococcal concentration (log_10 _cfu mL^-1 ^± SD; n = 3)	Identified species (number of isolates)	Number of PFGE profiles (*S. epidermidis*)	Characterized *S. epidermidis *strains
**A. Women with mastitis**				

1	5.28 ± 0.05	*S. epidermidis *(5) *S. aureus *(5)	1	C213

2	4.78 ± 0.32	*S. epidermidis *(10)	3	CJBP1 CJBP2 CJBP3

3	Nd	-	-	-

4	Nd	-	-	-

5	4.91 ± 0.29	*S. epidermidis *(8) *S. aureus *(1) *S. pasteuri *(1)	1	B

6	4.41 ± 0.17	*S. epidermidis *(7) *S. hominis *(3)	1	K

7	4.04 ± 0.09	*S. epidermidis *(7) *S. aureus *(3)	2	CJ9 CJ11

8	4.91 ± 0.50	*S. epidermidis *(10)	3	S1LDC12 S1LDC13 S1LDC18

9	4.72 ± 0.44	*S. epidermidis *(2) *S. pasteuri *(4) *S. hominis *(4)	1	F12

10	4.23 ± 0.47	*S. epidermidis *(10)	1	DC2Lae

11	4.38 ± 0.22	*S. epidermidis *(6) *S. aureus *(4)	1	B1CD2

12	4.08 ± 0.51	*S. epidermidis *(10)	1	DD2Laa

13	Nd	-	-	-

14	4.25 ± 0.08	*S. epidermidis *(5) *S. aureus *(5)	1	PLD21

15	4.41 ± 0.15	*S. epidermidis *(10)	1	P2LD1

16	4.51 ± 0.12	*S. epidermidis *(6) *S. warneri *(4)	1	M121

17	4.52 ± 0.04	*S. epidermidis *(7) *S. pasteuri *(3)	1	DF2Lab

18	4.80 ± 0.53	*S. epidermidis *(8) *S. warneri *(2)	1	V1LD1

19	5.68 ± 0.22	*S. epidermidis *(8) *S. pasteuri *(2)	1	DH3LIk

20	4.48 ± 0.33	*S. epidermidis *(9) *S. hominis *(1)	2	DG2ñ DG2s

21	4.04 ± 0.12	*S. epidermidis *(5) *S. warneri *(5)	1	YGLI4

22	4.17 ± 0.06	*S. epidermidis *(7) *S. aureus *(3)	1	ASLI3

23	5.44 ± 0.09	*S. epidermidis *(10)	3	ASLD1 ASLD2 ASLD3

24	4.15 ± 0.45	*S. epidermidis *(7) *S. pasteuri *(3)	1	ARLI1

25	4.64 ± 0.14	*S. epidermidis *(10)	4	Z2LDC11 Z2LDC12 Z2LDC14 Z2LDC17

26	4.02 ± 0.22	*S. epidermidis *(10)	1	AQLI2

27	4.05 ± 0.07	*S. epidermidis *(6) *S. aureus *(4)	1	AQLD3

28	4.04 ± 0.03	*S. aureus *(10)	-	-

29	4.09 ± 0.09	*S. epidermidis *(7) *S. pasteuri *(3)	1	AEA1

30	4.05 ± 0.24	*S. epidermidis *(10)	4	YLIC13 YLIC14 YLIC16 YLIC17

**B. Healthy women**				

1	2.91 ± 0.27	*S. epidermidis *(5) *S. aureus *(4) *S. lugdunensis *(1)	5	LC016 LC017 LC019 LC044 LC047

2	2.41 ± 0.09	*S. epidermidis *(10)	3	LE010 LE011 LE035

3	2.04 ± 0.11	*S. epidermidis *(10)	5	LG005 LG006 LG5021 LG5022 LG5023

4	1.91 ± 0.12	*S. epidermidis *(10)	2	LP22 LP223

5	2.02 ± 0.29	*S. epidermidis *(8) *S. hominis *(2)	3	LV221 LV222 LV521

6	2.93 ± 0.21	*S. epidermidis *(10)	3	LX5RB3 LX5RB4 LX5081

7	2.38 ± 0.14	*S. epidermidis *(4) *S. aureus *(4) *S. hominis *(2)	3	LO5081 LO5082 LO5RB1

8	2.58 ± 0.31	*S. epidermidis *(10)	3	LCC5081 LCC5082 LCC0592

9	2.48 ± 0.07	*S. epidermidis *(8) *S. aureus *(2)	4	LI5081 LI5094 LIRB1 LIRB2

10	2.25 ± 0.10	*S. epidermidis *(10)	2	LV5081 LV5RB3

11	2.41 ± 0.12	*S. epidermidis *(10)	2	LG5082a LGRB1

12	2.51 ± 0.22	*S. epidermidis *(10)	1	24C13

### Genotyping of the *S. epidermidis *isolates by PFGE profiling

**Figure 1:**
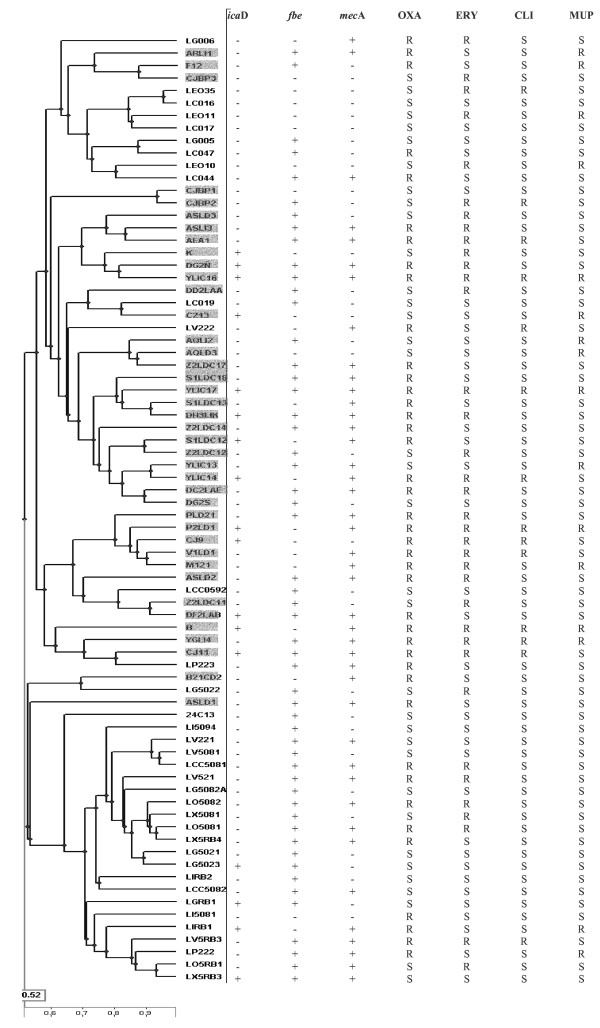
**Dendogram obtained by PFGE analysis of the *S. epidermidis *strains from mastitis and healthy women**. Strains isolated from mastitis-suffering women are shaded. Information on the presence of *ica*D, *fbe *and *mec*A genes and resistance to oxacillin (OXA; MIC > 2 μg mL^-1^), erythromycin (ERY; MIC > 4 μg mL^-1^), clindamycin (CLY; MIC > 2 μg mL^-1^) and mupirocin (MUP; MIC > 512 μg mL^-1^) has been included.

The 200 isolates of *S. epidermidis *recovered in this study were subjected to PFGE genotyping together with 105 isolates previously obtained from breast milk of 12 healthy women (Table 1). The analysis of the fingerprints obtained revealed the existence of 40 different pulsotypes among the isolates from women with mastitis and 36 among healthy women. Comparison of these genotypes showed that most of the strains grouped together depending on their origin in two different clusters, one containing most of the strains obtained from mastitic milk while the second contained most of the strains isolated from milk of healthy women (Figure 1).

### Detection of virulence determinants among the *S. epidermidis *strains

The 76 different *S. epidermidis *strains (40 from milk of women with mastitis and 36 strains from that of healthy women) were selected to study the presence of potential virulence traits. Hemolytic activity could not be detected or was very weak among all the assayed strains. In relation to adhesion-related genes, the multiplex PCR assay revealed the presence of the genes *embp *and *atl*E in all the strains. The *fbe *gene was detected in 65% of the strains from mastitis and in 75% of those isolated from healthy women (P = 0,3434). In contrast, the *ica*D gene was more prevalent among strains from mastitis cases (33%) than in those from healthy women (11%) (P = 0,0255) (Figure 1). A good correlation was observed between the presence of biofilm-related *ica *operon and the results obtained using the CRA assay, which determines potential for slime production, and all the strains that amplified for the gene gave also positive results by the phenotypic assay.

### Determination of MIC's to several antibiotics

Determination of MIC's to 21 antibiotics or antibiotics mixtures in the 76 *S. epidermidis *strains revealed that all of them were susceptible to the lower concentration of nitrofurantoin (32 μg mL^-1^) and rifampin (1 μg mL^-1^) while the results against the rest of antibiotics were variable depending on the strains (Table 2). Independently of their origin, most of the strains were sensitive to trimethoprim/sulfamethoxazole (MIC < 2/38 μg mL^-1 ^for 90% of the strains), gentamicyn (≤ 2 μg mL^-^1 for 87%), linezolid (≤ 2 μg mL^-1 ^for 86%), fosfomicyn (≤ 16 μg mL^-1 ^for 82%), ciprofloxacin (≤ 0,5 μg mL^-1 ^for 76%), tetracycline (≤ 8 μg mL^-1 ^for 75%), chloramphenicol (≤ 16 μg mL^-1 ^for 90%), penicillin (≤ 4 μg mL^-1 ^for 72%), ampicillin (≤ 4 μg mL^-1 ^for 80%) and the glycopeptides vancomycin (≤ 2 μg mL^-1 ^for 93%) and teicoplanin (≤ 1 μg mL^-1 ^for 70%). The percentage of susceptible strains was lower for imipenem (≤ 0,12 μg mL^-1^for 58%) and quinupristin/dalfopriscin (≤ 0,25 μg mL^-1 ^for 57%). However, significant differences were observed in the percentage of strains resistant to some antibiotics depending on their origin (Figure 1). For instance, 43% of isolates from mastitic samples showed a MIC of mupirocin ≥ 512 μg mL^-1 ^while only 22% of those isolated from non-mastitic samples reached this value (P = 0,0437). Similarly, 60% of the mastitic-related strains showed a MIC > 4 μg mL^-1 ^against erythromycin in contrast to 33% of the other group (P = 0,0201). In the case of clindamycin, 28% of the strains from mastitic milk presented a MIC > 2 μg mL^-1 ^while the percentage was of 8% in strains from healthy women (P = 0,0314). A higher percentage of oxacillin resistance (MIC > 2 μg mL^-1^) was also found among the strains from mastitis (68%) than those from healthy mothers (39%) (P = 0,0125). Finally, two strains from women with mastitis (CJ11 and DG2S) were resistant to streptomycin (> 1000 μg mL^-1^) and one strain (AQLI2) from the same group was resistant to vancomycin (16 μg mL^-1^). No strains resistant to these two antibiotics were found among the strains from healthy women.

**Table 2:** Distribution of MIC's to several antibiotics among *S. epidermidis *isolated from mastitis and healthy women

			Percentage of strains for which the MIC (μg mL^-1^) was as follows:									
			
Antibiotics	Breast milk	N° of strains	≤ 0.03	0.12	0.25	0.5	1	2	4	8	> 8	
PEN	H	36	17	8		8	14	14	8	11	19	
	M	40	10	5	5	8	10	33	8	8	18	

AMP				≤ 0.12	0.25	0.5	1	2	4	8	100	> 100
			
	H	36		19	6	17	22	8	8	6	14	
	M	40		15	8	5	23	20	10	5	13	3

OXA					≤ 0.25	0.5	1	2	> 2			
			
	H	36			11	31	11	8	**39**			
	M	40			5	8	13	8	**68**			

CIP					≤ 0.25	0.5	1	2	> 2			
			
	H	36			47	39	8		6			
	M	40			30	38	18	3	13			

CHL										≤ 8	16	> 16
			
	H	36								75	17	8
	M	40								78	10	13

ERY					≤ 0.25	0.5	1	2	4	> 4		
			
	H	36			39	14	6	8		**33**		
	M	40			23	15			3	**60**		

CLI						≤ 0.5	1	2	> 2			
			
	H	36				81	8	3	**8**			
	M	40				70	3		**28**			

TET									≤ 4	8	> 8	
			
	H	36							56	19	25	
	M	40							68	8	25	

VAN						≤ 0.5	1	2	4	8	16	≥ 16
			
	H	36					44	50	6			
	M	40					43	48	5	3	3	

MUP									≤ 4	256	> 256	
			
	H	36							78	11	**11**	
	M	40							58	13	**30**	

H: strains isolated from healthy women; M: strains isolated from mastitis-suffering women; PEN: penicillin; AMP: ampicillin; OXA: oxacillin; CIP: ciprofloxacin; CHL: chloramphenicol; ERY: erythromycin; CLI: clindamycin; TET: tetracycline; VAN: vancomycin; MUP: mupirocin.

Statistically-significant differences between isolates from mastitis and healthy women are in bold.

### Presence of *mecA *and SCC*mec *typing

Among the 41 strains showing oxacillin resistance, the *mec*A gene could be detected by PCR in 37 (25 from mastitic milk and 12 from milk of healthy women). No amplification was observed in two strains of each group (F12 and CJ9; LI5081 and LC047, respectively), which had shown an oxacillin MIC value of > 2 μg mL^-1^. In contrast, the *mec*A gene was detected in five oxacillin susceptible strains, one from a mastitis case (YLIC13) and four from healthy women (LO5RB1, LX5RB3, LV221 and LCC5082).

The type of SCC*mec *was determined in all the *mec*A^+ ^strains. The *ccr*B gene could be amplified from 22 of the 26 *mec*A^+ ^strains from the mastitis group and, on the basis of the *ccr*B restriction pattern with *Hinf*I (type IV: 264, 227 and 154 bp; type III: 537 and 106 bp) or with *Hinf*I/*Bsm*I (type IV: 227, 171, 153 and 93 bp; type III: 320, 174, 106 and 44 bp), 19 strains were assigned to type IV and the remaining three (S1LDC12, Z2LDC17 and DF2LAB) to type III (see additional file 1). Amplification of *ccr*B was achieved in the 16 *mec*A^+ ^strains from healthy women and, among them, 14 strains showed type IV and two type III SCC*mec*. The application of the full assay described by Zhang et al. [12] confirmed these results. The 4 *mecA*^+ ^strains that could not be typed on the basis of their *ccr*B restriction pattern remained non typeable with the full Zhang et al. assay.

## Discussion

*S. epidermidis *is a normal inhabitant of the skin and mucosal surfaces in healthy hosts and its ubiquitous prevalence as a commensal species makes difficult for a clinician to decide if an isolate represents the etiological agent or a culture contamination [11]. Therefore, isolation of this bacterial species is generally regarded as non-related to a mastitis case, even when it is the only species present in a milk sample [4].

*S. epidermidis *was the dominant or unique staphylococcal species in breast milk of women suffering mastitis, a finding described previously [4], which indicates that this bacterial species could be an etiological agent of human lactational mastitis. Similarly, several studies have shown the implication of this bacterial species as an agent of mastitis in different animal species [13-15].

The genotyping of the *S. epidermidis *isolates by PFGE revealed a low diversity within this species in the breast milk of women with mastitis, with a mean of 2 different profiles per sample. A lost in the microbial diversity present in milk of women with mastitis has been previously pointed [4]. Comparison by PFGE dendogram analysis of these strains with those obtained from breast milk of healthy women showed the existence of two main clusters and, within these two groups, the strains generally matched with the origin (mastitis and healthy women). However, a few strains from healthy women grouped together with the mastitis cluster reflecting a similar genetic background. The fact that their presence in milk of healthy women does not constitute a health problem could indicate that a predisposal host is also need for *S. epidermidis *to develop an infection [16].

Among the *S. epidermidis *strains analyzed, the presence of adhesion-related genes was high independently of the condition of the women from which they were isolated. The presence of genes encoding cell surface proteins may explain, at least partially, the high prevalence of *S. epidermidis *in breast milk, mammary areola and ducts of both healthy and mastitis-suffering women. In contrast, the percentage of strains carrying the biofilm-related *ica *operon was higher in strains from mastitis milk than in that obtained from healthy women. A potential relationship between *S. epidermidis *infection and the presence of such operon has been reported [8]. In fact, biofilm formation has been described in many cases of staphylococcal mastitis and this is the reason why such property is considered as a potential virulence factor [17,18].

Strains belonging to either the mastitis or the healthy women group showed a similar susceptibility against many of the antibiotics tested; however, a higher percentage of mupirocin-, erythromycin-, clindamicyn- and oxacillin-resistant strains was found among those isolated from mastitic milk. Resistance to these and other antibiotics in pathogenic *S. epidermidis *isolates has been reported previously [10,19]. The resistance of these strains could be partly due to the increasing use of broad-spectrum antibiotics, which encourage selection of multirresistant strains [11]. Improper antibiotherapy may explain why staphylococcal mastitis frequently becomes a chronic and/or recurrent infection. In this study, the presence of *mec*A gene accompanied with resistance to oxacillin (MIC > 2 μg mL^-1^) was observed in 62% of the strains from mastitis, but only in 33% from the healthy group. The *mec*A gene was not detected in four oxacillin-resistant strains. These strains may represent cases of borderline resistance which is characterized by an oxacillin MIC at or just above the susceptibly breakpoint (4 to 8 μg mL^-1^). In contrast, the *mec*A gene was detected in five oxacillin-susceptible strains, a fact that has been previously described [20] and that may be due to gene deletions.

Methicillin-resistant *Staphylococcus aureus *(MRSA) are being reported with increasing frequency in the community and they have been called community-acquired (CA)-MRSA, which are associated with skin and soft tissue infection [21] but are also frequently isolated from healthy hosts [22]. Most of the *mec*A^+ ^strains used in this study could be ascribed to type IV SCC*mec*. In *S. epidermidis*, some studies have reported that SCC*mec *type IV is generally carried by CA-MRS [23,24] but this type seems to be predominant among clinically relevant *S. epidermidis *isolates [9]. The fact that the *ccr*B gene was not amplified from four *mec*A^+ ^strains may be due to the presence of different alleles for this gene [25].

In the last years, a renewed medical and research interest has been focused on *S. epidermidis *since it has become the most important cause of nosocomial infections [6]. The complete genome analysis of some methicillin-resistant *S. aureus *and *S. epidermidis *strains of human origin have revealed the propensity of *S. aureus *to cause fulminant and sometimes life-threatening infections, as opposed to the predisposition of *S. epidermidis *for chronic and recurrent infections [26]. Identification of *S. epidermidis *as etiological agents of infection is sometimes hindered by the fact that infections associated with this microorganims are characterized by subtle, non-specific clinical manifestations [5]. Precisely, these characteristics occur in most cases of lactational mastitis. Genome flexibility in *S. epidermidis *may contribute to the acquisition of some transferable virulence and resistant traits [6,27] and to the evolution of this species from a commensal to a pathogenic microorganism in susceptible hosts [28]. In fact, it seems that there is an important host factor in lactational mastitis since a woman having such condition usually displays the same clinical signs after subsequent pregnancies. Additionally, it would explain why only a 3–30% of lactating women suffer from such infection when it is the predominant bacterial species found in breast milk of healthy women [29,30].

## Conclusion

*Staphylococcus epidermidis *is the most prevalent staphylococcal species isolated from breast milk of women suffering mastitis, where it is present at a concentration notably higher than that present in milk of healthy woman (≥ 4.0 versus ≤ 3.0 log_10 _cfu mL^-1^, respectively). The percentage of strains showing biofilm production ability and resitance to mupirocin, erythromycin, clindamicyn and/or methicillin was significantly higher among those obtained from women with lactational mastitis than among those isolated from healthy women.

The random method used to select staphylococcal colonies from the samples could introduce a bias regarding the low number of samples from which *S. aureus *was isolated. Traditionally, *S. aureus *has been considered as the main etiological agent of mastitis. However, the results of this work suggest that *S. epidermidis *could be an additional and underrated cause of lactational mastitis; as a consequence, its presence should be also considered in bacteriological analyses of human milk when there is a suspicious of a mastitis infection. Further studies involving a larger number of samples and staphylococcal isolates will be required to confirm the results obtained in this study.

## Methods

### Samples and isolation of staphylococcal isolates

A total of 30 women aged 25–34 years with clinical symptoms of infectious mastitis participated in the study (Table 1). They were diagnosed by the lactation consultants attending different primary health-care centers in Spain in a 2-months period (October-November 2007). The total staphylococcal count was higher ≥ 4 log_10 _cfu mL^-1 ^in all their samples. Women with mammary abscesses or any kind of parallel diseases and patients treated with antibiotherapy during the previous two weeks of the study were excluded. All volunteers gave written informed consent to the protocol, which was approved by the Ethical Committee of Hospital Clínico of Madrid (Spain). The milk samples were collected as described previously [31], and plated onto ready to use Baird Parker (BP) plates supplied by bioMérieux (Marcy l'Etoile, France). The plates were incubated in aerobiosis at 37°C for 48 h.

### Identification of staphylococci

Initially, a total of 270 isolates (10 from each sample displaying bacterial growth on BP plates) were randomly selected and tested for catalase and coagulase activities and for their resistance to lysozyme and lysostaphin [32]. All of them were subjected to a novel multiplex PCR method designed to allow a rapid identification of *S. epidermidis *and *S. aureus *isolates. The new primers (see below) were designed on the basis of the variable regions of the *tuf *gene sequence of *Staphylococcus *using the program Clone Manager Suite 7.0 (Sci Ed Central, USA). Their specificity was tested *in silico *by PCR simulation against up-to-date sequenced prokaryotic genomes using the tools provided in the website http://insilico.ehu.es/PCR. Briefly, a colony of each isolate was suspended in 20 μL of TE buffer (10 M Tris-HCl, 1 M EDTA pH 8) with 0.9% NaCl, and after heating at 98°C for 10 min, the suspension (5 μL) was used as a template for PCR using the BioredMix (BioLine, London, UK) system and the primers (10 μM) tuf-g (5'-GGTGTACCAGCATTAGT-3'), tuf-a (5'-TTCAGTATGTGGTGTAA-3') and tuf-e (5'-TTCGTGCATACCGATGA-3'). The primer pairs tuf-g/tuf-e and tuf-g/tuf-a result in a 370 bp (*S. epidermidis*) or a 530 bp (*S. aureus*) fragment [see additional file 2]. PCR conditions were 1 cycle of 94°C for 5 min, 30 cycles of 94°C for 1 min, 48°C for 1 min, and 72°C for 2 min, and a final extension of 72°C for 5 min. Identification of the isolates was confirmed by PCR sequencing of a 470 bp fragment of the 16S rRNA gene using primers and conditions previously described [33]. The amplicons were purified using the Nucleospin^® ^Extract II kit (Macherey-Nagel, Düren, Germany) and sequenced at the Genomics Unit of the Universidad Complutense de Madrid, Spain.

### Genotyping of *S. epidermidis *isolates by pulsed field gel electrophoresis (PFGE)

To determine the diversity of *S. epidermidis *in breast milk in mastitis infections, 200 isolates of this species obtained from 26 women with mastitis were subjected to PFGE genotyping together with 105 isolates of the same species obtained from breast milk of 12 healthy women within the same period of time [34] (Table 1). Chromosomal DNA was digested with the endonuclease *Sma*I (New England Biolabs, Ipswich, MA) at 37°C for 16 h. Electrophoresis was carried out in a CHEF DR-III apparatus (Bio-Rad Laboratories, Hercules, CA) for 23 h at 14°C at 6 V cm^-1 ^with pulses from 5 to 50 s. A standard pattern (Lamda Ladder PFG Marker, New England Biolabs) was included in the gels to allow comparison of the digitally normalized PFGE profiles. Computer-assisted analysis of the gels was performed with the Phoretix 1D Pro software (Nonlinear USA, Inc., Durham, NC). PFGE profiles differing in one or more fragments were considered different. Cluster analysis of the PFGE patterns was performed using the UPGMA method based on the Dice similarity coefficient.

### Screening for potential virulence determinants

On the basis of the different PFGE profiles, 76 strains (40 from mastitis cases and 36 from healthy women) were further selected and characterized. Presence of genes *embp*, *fbe*, *atl*E and *ica*D, which respective products are involved in adhesion and biofilm formation, was evaluated using primers couples described previously [7,35-37]. In the case of *fbe*, *atl*E and *ica*D, a multiplex PCR format was designed using the following conditions: 5 min at 94°C followed by 30 cycles of 94°C for 1 min, 60°C for 30 s, 72°C for 1 min and, then, a final extension of 5 min at 72°C [see additional file 3]. Strains isolated from mastitic milk were compared to those isolated from healthy women. Data were subjected to a statistical analysis using the Chi-square test (SPSS package, SPSS Inc, Chicago, IL, USA). Differences were considered significant if P values were lower than 0.05.

### Phenotypic assays

The hemolytic activity of the isolates was determined on Columbia agar supplemented with 5% horse blood (COH, bioMériux) after incubation at 37°C for 72 h following a procedure previously described [32]. The ability of the isolates to form slime was assessed using the Congo Red agar assay (CRA) [38]. The plates were incubated at 37°C for 24 h and, then, for additional 24 h at room temperature.

### Determination of MIC's to antibiotics

The determination of the MIC's to several antibiotics commonly used against staphylococcal infections was evaluated by a microdilution method using the Sensititre plates Staenc1F (Trek Diagnostic Systems, Cleveland, OH) following the manufacturer's instructions. The antibiotics analyzed were: penicillin, ampicillin, amoxycillin-clavulanic acid, teicoplanin, chloramphenicol, erythromycin, mupirocin, streptomycin, gentamicin, clindamycin, oxacillin, ciprofloxacin, fosfomycin, imipenem, nitrofurantoine, trimethoprim-sufamethoxazole, tetracycline, vancomycin, linezolid, quinupristin-dalfopristin and rifampin. Data were submitted to the statistical analysis described above.

### Screening for *mec*A gene and typing of the staphylococcal chromosome cassette *mec *(SSC*mec*)

Presence of the *mec*A gene was evaluated by PCR using primers *mec*A forward (5'-GGTCCCATTAACTCTGAAG-3') and *mec*A reverse (5'-AGTTCTGCAGTACCGGATTTTGC-3'), which results in a 1,040 bp fragment [39]. The SCC*mec *was subjected to a typing procedure [40], which implied the PCR amplification of the *ccr*B gene followed by RFLP analysis using endonucleases *Hinf*I and *Bsm*I. Presence of *mecA *and SCC*mec *typing was confirmed using all the primers and conditions described by Zhang et al. [12].

## Authors' contributions

SD carried out the microbiological analysis of the samples, designed the primers and multiplex PCR conditions and drafted the manuscript. RA assisted in the preparation of material and in the identification of the isolates. EJ and MLM participated in the characterization of the strains. RC set up and helped with the PFGE methodology. LF participated in the design of the study and performed the statistical analysis. JMR conceived of the study, coordinated it and revised the manuscript. All authors read and approved the final manuscript.

## Supplementary Material

Additional file 1 **PCR-RFLP of the *ccr*B gene using endonucleases *Hinf*I and *Hinf*I/*Bsm*I. The figure provided shows the profiles of SCC*mec *types III and IV using the method of Yang et al**. [40]. In lanes 1 and 3 *ccr*B amplicons are cut with *Hinf*I whereas in lanes 2 and 4 the amplicons are cut with *Hinf*I and *Bsm*I. Lanes 1 and 2: *S. epidermidis *DF2LAB, SCC*mec *type III (537, 106 bp and 320, 174, 106 bp respectively); lanes 3 and 4: *S. epidermidis *V1LD1, SCC*mec *type IV (264, 227, 154 and 227, 171, 153, 93 bp respectively); M, molecular weight marker. Click here for file

Additional file 2 **Multiplex *tuf *gene-based PCR assay for the specific identification of *S. aureus *and *S. epidermidis***. The figure provided shows the respective species-specific bands. Lanes: 1–5, *S. aureus *isolates; 6–9, *S. epidermidis *isolates, 10, negative control; M, molecular weight marker (100 bp Ladder, Invitrogen). Click here for file

Additional file 3 **Multiplex PCR assay for the simultaneous detection of three adhesion- or biofilm-related genes**. The figure provided shows the respective gene-specific bands. Lanes: 1, *S. epidermidis *CJBP2; 2, *S. epidermidis *V1LD1; 3, *S. epidermidis *DG2S; 4, *S. epidermidis *P2LD1; 5, *S. epidermidis *S1LDC13; 6, negative control; M, molecular weight marker. *atl*E gene: 682 bp; *fbe *gene: 496 bp; *ica*D gene: 225 bp. Click here for file
