# Supplementary figures and images for: Staphylococcus epidermidis strains isolated from breast milk of women suffering infectious mastitis: potential virulence traits and resistance to antibiotics
Source: BMC Microbiol. 2009 May 7;9:82. doi: 10.1186/1471-2180-9-82 (PMC2685400; doi:10.1186/1471-2180-9-82)

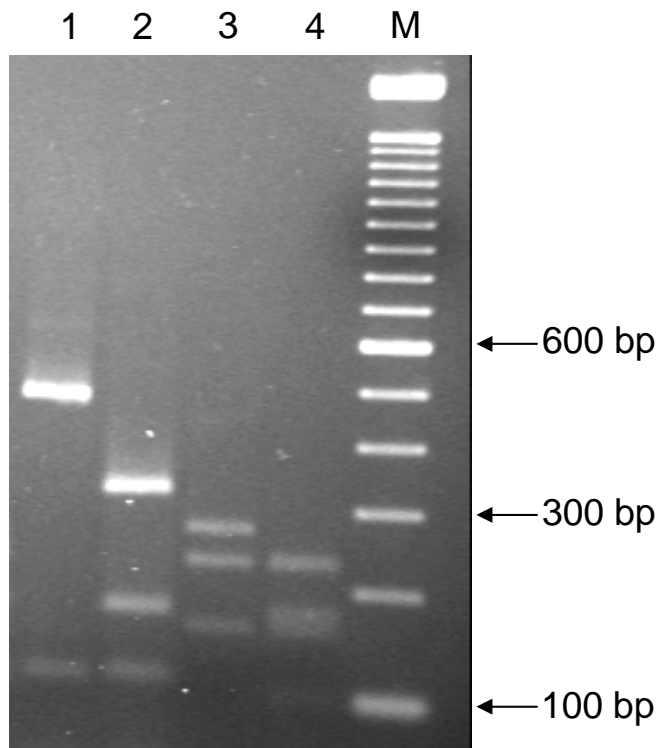

Supplement: Additional file 1 — PCR-RFLP of the ccrB gene using endonucleases HinfI and HinfI/BsmI. The figure provided shows the profiles of SCCmec types III and IV using the method of Yang et al. [40]. In lanes 1 and 3 ccrB amplicons are cut with HinfI whereas in lanes 2 and 4 the amplicons are cut with HinfI and BsmI. Lanes 1 and 2: S. epidermidis DF2LAB, SCCmec type III (537, 106 bp and 320, 174, 106 bp respectively); lanes 3 and 4: S. epidermidis V1LD1, SCCmec type IV (264, 227, 154 and 227, 171, 153, 93 bp respectively); M, molecular weight marker. [file 1471-2180-9-82-S1.pdf]

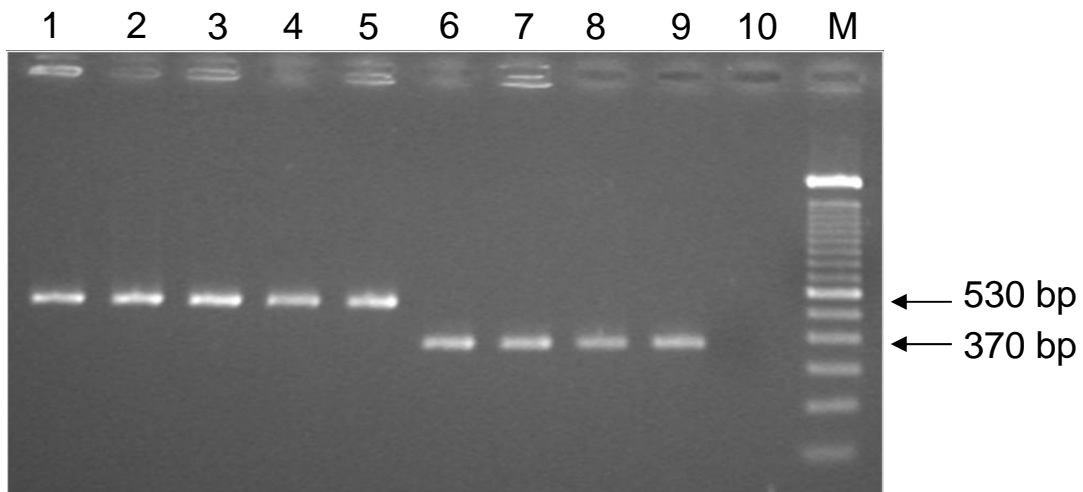

Supplement: Additional file 2 — Multiplex tuf gene-based PCR assay for the specific identification of S. aureus and S. epidermidis. The figure provided shows the respective species-specific bands. Lanes: 1–5, S. aureus isolates; 6–9, S. epidermidis isolates, 10, negative control; M, molecular weight marker (100 bp Ladder, Invitrogen). [file 1471-2180-9-82-S2.pdf]

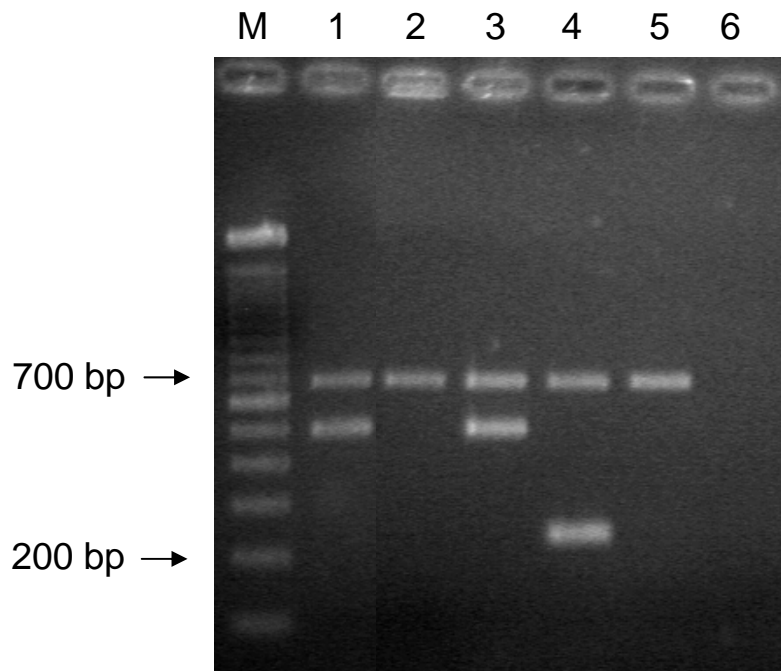

Supplement: Additional file 3 — Multiplex PCR assay for the simultaneous detection of three adhesion- or biofilm-related genes. The figure provided shows the respective gene-specific bands. Lanes: 1, S. epidermidis CJBP2; 2, S. epidermidis V1LD1; 3, S. epidermidis DG2S; 4, S. epidermidis P2LD1; 5, S. epidermidis S1LDC13; 6, negative control; M, molecular weight marker. atlE gene: 682 bp; fbe gene: 496 bp; icaD gene: 225 bp. [file 1471-2180-9-82-S3.pdf]
